# 1,12-Bis(2-carboxy­phen­yl)-5,8-dioxa-2,11-diaza­dodecane-2,11-diium dichloride methanol solvate

**DOI:** 10.1107/S1600536809051824

**Published:** 2009-12-04

**Authors:** Samuel Johnson, Ghezai T. Musie, Edward R. T. Tiekink

**Affiliations:** a Department of Chemistry, University of Texas at San Antonio, One UTSA Circle, San Antonio, TX 78249-0698, USA; b Department of Chemistry, University of Malaya, 50603 Kuala Lumpur, Malaysia

## Abstract

In the title salt hydrate, C_22_H_30_N_2_O_6_^2+^·2Cl^−^·CH_4_O, the dication adopts a U-shaped conformation whereby the benzene rings are splayed out from the chain linking them. All components of the asymmetric unit are linked into a cohesive entity by a combination of O—H⋯Cl^−^, N^+^—H⋯Cl^−^ and N^+^—H⋯O charge-assisted hydrogen-bonding inter­actions. The assemblies thus formed are linked into supra­molecular helical chains along [010] *via* C—H⋯O contacts. The resulting chains are, in turn, consolidated into the three-dimensional crystal structure by C—H⋯π contacts.

## Related literature

For related literature on dinucleating ligands, see: Fenton & Okawa (1997 ▶); Uhlenbrock & Krebs (1992 ▶); Ghiladi *et al.* (1997 ▶); Koga *et al.* (1998 ▶); Kitagawa *et al.* (2004 ▶); Bradshaw *et al.* (2005 ▶).
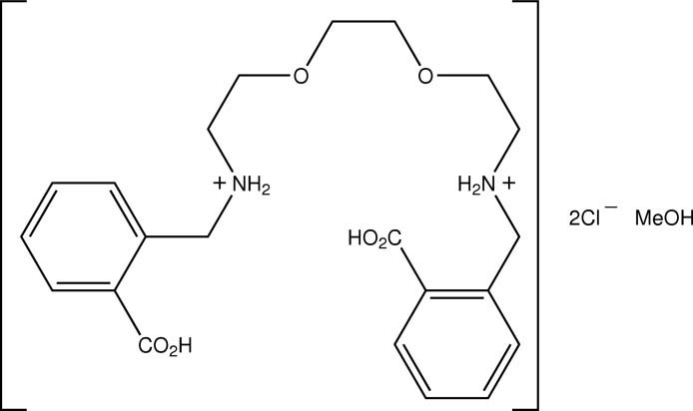


## Experimental

### 

#### Crystal data

C_22_H_30_N_2_O_6_^2+^·2Cl^−^·CH_4_O
*M* *_r_* = 521.42
Monoclinic, 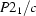

*a* = 11.567 (3) Å
*b* = 11.352 (2) Å
*c* = 20.052 (5) Å
β = 102.760 (6)°
*V* = 2568.0 (10) Å^3^
*Z* = 4
Mo *K*α radiation
μ = 0.30 mm^−1^
*T* = 93 K
0.40 × 0.30 × 0.20 mm

#### Data collection

Rigaku AFC12κ/SATURN724 diffractometer
Absorption correction: multi-scan (*ABSCOR*; Higashi, 1995 ▶) *T*_min_ = 0.824, *T*_max_ = 1.000
66801 measured reflections
5874 independent reflections
5598 reflections with *I* > 2σ(*I*)
*R*_int_ = 0.050
Standard reflections: 0

#### Refinement

*R*[*F* ^2^ > 2σ(*F* ^2^)] = 0.036
*wR*(*F* ^2^) = 0.094
*S* = 1.05
5874 reflections
316 parameters
3 restraints
H-atom parameters constrained
Δρ_max_ = 0.51 e Å^−3^
Δρ_min_ = −0.34 e Å^−3^

### 

Data collection: *CrystalClear* (Rigaku/MSC 2005 ▶); cell refinement: *CrystalClear*; data reduction: *CrystalClear*; program(s) used to solve structure: *SHELXS97* (Sheldrick, 2008 ▶); program(s) used to refine structure: *SHELXL97* (Sheldrick, 2008 ▶); molecular graphics: *DIAMOND* (Brandenburg, 2006 ▶); software used to prepare material for publication: *publCIF* (Westrip, 2009 ▶).

## Supplementary Material

Crystal structure: contains datablocks global, I. DOI: 10.1107/S1600536809051824/hg2615sup1.cif

Structure factors: contains datablocks I. DOI: 10.1107/S1600536809051824/hg2615Isup2.hkl

Additional supplementary materials:  crystallographic information; 3D view; checkCIF report

## Figures and Tables

**Table 1:** Hydrogen-bond geometry (Å, °)

*D*—H⋯*A*	*D*—H	H⋯*A*	*D*⋯*A*	*D*—H⋯*A*
O2—H2*O*⋯Cl1	0.84	2.20	3.0316 (13)	170
O6—H6*O*⋯Cl2	0.84	2.12	2.9602 (13)	177
O7—H7*O*⋯Cl1	0.84	2.32	3.1586 (19)	174
N1—H1*A*⋯O1	0.92	2.05	2.7729 (16)	135
N1—H1*A*⋯O3	0.92	2.25	2.6809 (16)	108
N1—H1*B*⋯Cl2	0.92	2.27	3.1110 (13)	151
N2—H2*A*⋯O4	0.92	2.32	2.7245 (16)	106
N2—H2*A*⋯O5	0.92	2.04	2.7678 (16)	135
N2—H2*B*⋯Cl1	0.92	2.25	3.1157 (13)	156
C14—H14*B*⋯O7^i^	0.99	2.46	3.273 (2)	139
C8—H8*B*⋯*Cg*^ii^	0.99	2.88	3.6364 (16)	134

Symmetry codes: (i) 
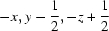
; (ii) 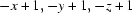. *Cg* is the centroid of the C16–C21 ring.

**Fig. 1.**
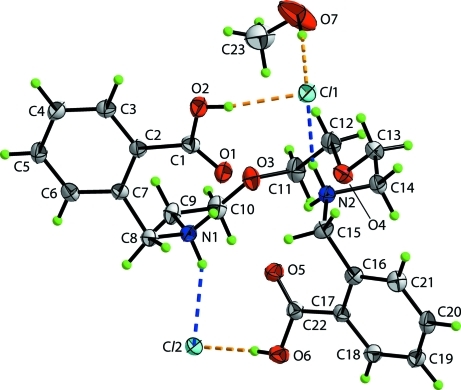
Molecular structure of the asymmetric unit in (I), showing atom-labelling scheme and displacement ellipsoids at the 50% probability level. The O–H···Cl- and N+–H···Cl- interactions are shown as orange and blue dashed lines, respectively.

**Fig. 2.**
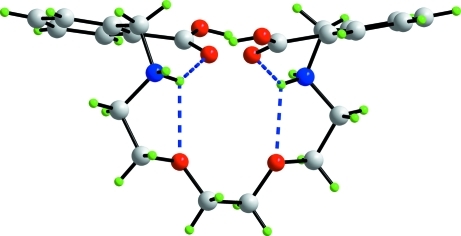
A view of the dication in (I) highlighting the U-shaped geometry and the N+–H···O hydrogen bonds (blue dashed lines). Color code: Cl, cyan; O, red; N, blue; C, grey; and H, green.

**Fig. 3.**
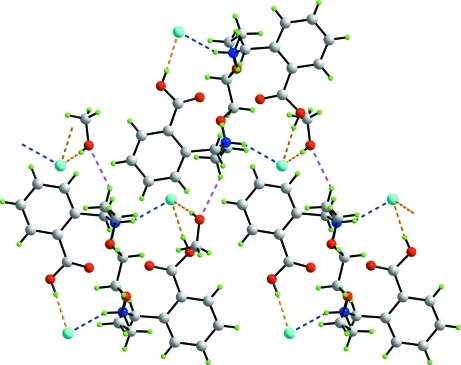
The supramolecular chains in (I) aligned along [0 1 0]. The O–H···Cl- and N+–H···Cl- hydrogen bonds are shown as orange and blue dashed lines, respectively. The C–H···O contacts are shown as pink dashed lines. Color code: Cl, cyan; O, red; N, blue; C, grey; and H, green.

**Fig. 4.**
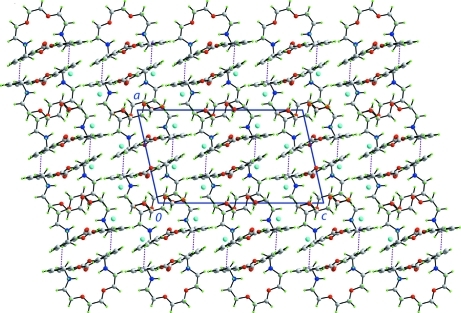
A view in projection down the b axis in (I) highlighting the connections between supramolecular chains along [0 1 0]. The C–H···π interactions are represented by purple dashed lines. Color code: Cl, cyan; O, red; N, blue; C, grey; and H, green.

## supplementary crystallographic information

### Comment

Since the concept of dinucleating ligands was developed (*e.g.* Fenton & Okawa, 1997; Uhlenbrock & Krebs, 1992; Koga *et al.*, 1998), various dinucleating ligands that stabilize dinuclear metal complexes have been reported in the literature (*e.g.* Ghiladi *et al.*, 1997; Kitagawa *et al.* 2004). Such ligands have also attracted attention in supramolecular and materials chemistry owing to their variety of structural topologies and potential applications in the rational design of metal-organic frameworks (Bradshaw *et al.*, 2005). Motivated by the above, the title compound, (I), was prepared as part of a on-going study aimed at developing this chemistry.

The crystallographic asymmetric unit of (I) comprises a 1,12-bis(2-carboxyphenyl)-5,8-dioxa-2,11-diazadodecane-2,11-diium dianion, two chloride anions, and a methanol molecule of solvation, Fig. 1. The central atoms, *i.e.* O3, O4, N1, N2 and C9–C14, define an approximate plane (r.m.s. for the fitted atoms = 0.239 Å), even allowing for the fact that the plane comprises only *sp*^3^ atoms. The C8 and C15 atoms lie 0.5197 (17) and 0.3720 (17) Å to either side of this plane, indicating that the benzene rings also lie above and below the central plane. The orientations of the benzene rings are orthogonal to the central plane which forms dihedral angles with the C2–C7 and C16–C21 benzene rings of 89.20 (4) and 86.67 (4) °, respectively. The benzene rings have similar orientations with respect to the central plane and form a dihedral angle of 20.17 (3) ° to each other. Overall, the conformation of the molecule is U-shaped with the benzene rings splayed out to either side as illustrated in Fig. 2.

As indicated in Figs 1 and 2, there are a large number of hydrogen bonding interactions in the structure, and these occur between species comprising the asymmetric unit, Table 1. Each of the carboxylic acid groups as well as the hydroxyl group of the solvent molecule forms a charge-assisted O–H···Cl^-^ hydrogen bond, Fig. 1. The ammonium groups behave similarly to each other in terms of forming hydrogen bonding interactions. The N1—H1a atom forms disparate N^+^–H···O hydrogen bonds with two carbonyl-O atoms, Fig. 2, and the H1b atom forms a charge-assisted N^+^–H···Cl^-^ hydrogen bond, Fig. 1. In the case of N1–H1b, the shorter N^+^–H···O interaction is formed with the carbonyl-O5 atom and the weaker contact is made with the ester-O4 atom, Fig. 2. The hydrogen bonding thus far described provides stability to the asymmetric unit.

The most prominent interactions linking the asymmetric units are of the type C–H···O, Table 1. These involve the solvent methanol molecule which in turn is connected to both the Cl1 anion and the carboxylic acid-O2 atom of a second cation. In this fashion a supramolecular helical chain is formed along [0 1 0], Fig. 3. Fig. 3 clearly illustrates the differences between the chlorides in that the Cl1^-^ anion forms three significant hydrogen bonding contacts and the Cl2^-^ atom only forms two. The supramolecular chains are consolidated into the crystal structure *via* C–H···π interactions, Fig. 4 and Table 1. These occur between methylene-C8–H8b and the ring centroid, *Cg*, of the C16–C21 ring so that H8b···*Cg*^i^ = 2.88 Å, C8···*Cg*^i^ = 3.6364 (16) Å with an angle of 134 ° subtended at the H8b atom, for symmetry operation i: 1 - *x*, 1 - *y*, 1 - *z*.

### Experimental

In a 200 ml round bottom flask, 2-carboxybenzaldehyde (4.0424 g, 0.027 mol) was dissolved in methanol (30 ml). While stirring at 308 K, sodium hydroxide (1.0754 g, 0.027 mol) was added. In a separate flask, 2,2'-(ethylenedioxy)bis(ethylamine) (NOON) (1.951 ml, 1.98 g, 0.013 mol) was dissolved in methanol (10 ml). The NOON solution was added drop-wise into the original solution using a pipette. The reaction was left to react for 2.5 h. NaBH_4_ (2.4296 g, 0.0643 mol) was then added to the flask in small portions. The resultant mixture was heated and stirred for another 2.5 h. It was then cooled in an ice bath and filtered to remove any solid. To the filtrate, concentrated HCl was added drop wise until the pH was about 2–3. A white precipitate was formed and filtered using gravity filtration. The white product was washed with acetone three times to remove any impurity and was dried. ^1^H NMR (D_2_O): δ 8.17 (s, 1H, aldamine-H), 7.75 (d, 2H, Ar—H), 7.60 (d, 2H, Ar—H), 7.41 (t, 2H, Ar—H), 7.36 (t, 2H, Ar—H), 3.87 (t, 2H, al-H), 3.75 (s, 2H, al-H), 3.45 (t, 2H, al-H) p.p.m.

Single crystals were obtained by slow evaporation of an acidic (1*M* HCl) aqueous solution of (I).

### Refinement

The N- and C-bound H atoms were geometrically placed (N—H = 0.92 Å and C—H = 0.95–0.99 Å) and refined as riding with *U*_iso_(H) = 1.2–1.5*U*_eq_(C). The O—H hydrogen atoms were located from a difference map and refined with O—H = 0.84 (1) Å, and with *U*_iso_(H) = 1.*5U*_eq_(O).

### Crystal data

**Table:**

C_22_H_30_N_2_O_6_^2+^·2Cl^−^·CH_4_O	*F*(000) = 1104
*M**_r_* = 521.42	*D*_x_ = 1.349 Mg m^−^^3^
Monoclinic, *P*2_1_/*c*	Mo *K*α radiation, λ = 0.71073 Å
Hall symbol: -P 2ybc	Cell parameters from 10052 reflections
*a* = 11.567 (3) Å	θ = 2.3–27.5°
*b* = 11.352 (2) Å	µ = 0.30 mm^−^^1^
*c* = 20.052 (5) Å	*T* = 93 K
β = 102.760 (6)°	Needle, pale-yellow
*V* = 2568.0 (10) Å^3^	0.40 × 0.30 × 0.20 mm
*Z* = 4	

### Data collection

**Table:**

Rigaku AFC12κ/SATURN724 diffractometer	5874 independent reflections
Radiation source: fine-focus sealed tube	5598 reflections with *I* > 2σ(*I*)
graphite	*R*_int_ = 0.050
ω scans	θ_max_ = 27.6°, θ_min_ = 2.4°
Absorption correction: multi-scan (*ABSCOR*; Higashi, 1995)	*h* = −15→15
*T*_min_ = 0.824, *T*_max_ = 1.000	*k* = −14→14
66801 measured reflections	*l* = −25→23

### Refinement

**Table:**

Refinement on *F*^2^	Primary atom site location: structure-invariant direct methods
Least-squares matrix: full	Secondary atom site location: difference Fourier map
*R*[*F*^2^ > 2σ(*F*^2^)] = 0.036	Hydrogen site location: inferred from neighbouring sites
*wR*(*F*^2^) = 0.094	H-atom parameters constrained
*S* = 1.05	*w* = 1/[σ^2^(*F*_o_^2^) + (0.0485*P*)^2^ + 1.3403*P*] where *P* = (*F*_o_^2^ + 2*F*_c_^2^)/3
5874 reflections	(Δ/σ)_max_ = 0.001
316 parameters	Δρ_max_ = 0.51 e Å^−^^3^
3 restraints	Δρ_min_ = −0.34 e Å^−^^3^

### Special details

**Table:**

Geometry. All s.u.'s (except the s.u. in the dihedral angle between two l.s. planes) are estimated using the full covariance matrix. The cell s.u.'s are taken into account individually in the estimation of s.u.'s in distances, angles and torsion angles; correlations between s.u.'s in cell parameters are only used when they are defined by crystal symmetry. An approximate (isotropic) treatment of cell s.u.'s is used for estimating s.u.'s involving l.s. planes.
Refinement. Refinement of *F*^2^ against ALL reflections. The weighted *R*-factor *wR* and goodness of fit *S* are based on *F*^2^, conventional *R*-factors *R* are based on *F*, with *F* set to zero for negative *F*^2^. The threshold expression of *F*^2^ > 2σ(*F*^2^) is used only for calculating *R*-factors(gt) *etc*. and is not relevant to the choice of reflections for refinement. *R*-factors based on *F*^2^ are statistically about twice as large as those based on *F*, and *R*- factors based on ALL data will be even larger.

### Fractional atomic coordinates and isotropic or equivalent isotropic displacement parameters (Å^2^)

**Table:**

	*x*	*y*	*z*	*U*_iso_*/*U*_eq_	
Cl1	0.17035 (3)	0.90851 (3)	0.294477 (16)	0.02137 (9)	
Cl2	0.38918 (3)	0.44293 (3)	0.645104 (15)	0.01982 (9)	
O1	0.28564 (9)	0.80955 (8)	0.46826 (5)	0.0219 (2)	
O2	0.31089 (9)	0.99194 (8)	0.43228 (5)	0.0226 (2)	
H2O	0.2788	0.9626	0.3942	0.034*	
O3	0.06032 (8)	0.70435 (9)	0.54842 (5)	0.0203 (2)	
O4	−0.00828 (8)	0.63200 (8)	0.41190 (5)	0.01964 (19)	
O5	0.29306 (9)	0.53733 (8)	0.47333 (5)	0.0206 (2)	
O6	0.37621 (9)	0.36208 (8)	0.50329 (5)	0.0218 (2)	
H6O	0.3793	0.3878	0.5429	0.033*	
O7	−0.03491 (14)	1.03638 (17)	0.34730 (7)	0.0617 (5)	
H7O	0.01604	1.0020	0.3302	0.093*	
N1	0.29662 (9)	0.68943 (9)	0.58991 (5)	0.0149 (2)	
H1A	0.2544	0.7080	0.5467	0.018*	
H1B	0.3051	0.6088	0.5919	0.018*	
N2	0.18404 (9)	0.65500 (9)	0.35526 (5)	0.0161 (2)	
H2A	0.1871	0.6387	0.4006	0.019*	
H2B	0.1845	0.7356	0.3507	0.019*	
C1	0.32087 (11)	0.91027 (11)	0.48045 (6)	0.0168 (2)	
C2	0.37852 (11)	0.95398 (11)	0.55001 (6)	0.0160 (2)	
C3	0.39169 (12)	1.07574 (11)	0.56072 (7)	0.0197 (3)	
H3	0.3632	1.1281	0.5238	0.024*	
C4	0.44551 (12)	1.12139 (12)	0.62416 (7)	0.0212 (3)	
H4	0.4538	1.2041	0.6306	0.025*	
C5	0.48703 (11)	1.04508 (12)	0.67811 (7)	0.0188 (3)	
H5	0.5249	1.0754	0.7216	0.023*	
C6	0.47325 (11)	0.92430 (11)	0.66853 (6)	0.0169 (2)	
H6	0.5010	0.8730	0.7061	0.020*	
C7	0.41960 (10)	0.87617 (11)	0.60510 (6)	0.0149 (2)	
C8	0.41731 (10)	0.74319 (11)	0.59949 (7)	0.0161 (2)	
H8A	0.4519	0.7201	0.5604	0.019*	
H8B	0.4684	0.7100	0.6415	0.019*	
C9	0.22530 (11)	0.72536 (11)	0.63977 (6)	0.0180 (2)	
H9A	0.2190	0.8123	0.6409	0.022*	
H9B	0.2640	0.6977	0.6862	0.022*	
C10	0.10344 (12)	0.67130 (12)	0.61791 (7)	0.0205 (3)	
H10A	0.1081	0.5845	0.6223	0.025*	
H10B	0.0504	0.7014	0.6466	0.025*	
C11	−0.05474 (11)	0.65894 (13)	0.52063 (7)	0.0223 (3)	
H11A	−0.1127	0.6933	0.5448	0.027*	
H11B	−0.0550	0.5723	0.5264	0.027*	
C12	−0.08839 (11)	0.68984 (13)	0.44601 (7)	0.0222 (3)	
H12A	−0.1706	0.6641	0.4264	0.027*	
H12B	−0.0840	0.7762	0.4401	0.027*	
C13	−0.03062 (11)	0.66044 (12)	0.34123 (7)	0.0196 (3)	
H13A	−0.0340	0.7469	0.3349	0.023*	
H13B	−0.1072	0.6264	0.3170	0.023*	
C14	0.06982 (11)	0.60919 (12)	0.31388 (7)	0.0191 (3)	
H14A	0.0684	0.5222	0.3167	0.023*	
H14B	0.0613	0.6317	0.2653	0.023*	
C15	0.29383 (11)	0.60681 (11)	0.33720 (7)	0.0179 (2)	
H15A	0.3636	0.6296	0.3731	0.022*	
H15B	0.3030	0.6422	0.2936	0.022*	
C16	0.29048 (10)	0.47434 (11)	0.33022 (6)	0.0155 (2)	
C17	0.30167 (10)	0.39592 (11)	0.38574 (6)	0.0154 (2)	
C18	0.29275 (11)	0.27445 (11)	0.37379 (7)	0.0175 (2)	
H18	0.2987	0.2220	0.4113	0.021*	
C19	0.27540 (11)	0.22952 (11)	0.30795 (7)	0.0190 (3)	
H19	0.2671	0.1471	0.3003	0.023*	
C20	0.27025 (11)	0.30619 (12)	0.25346 (7)	0.0196 (3)	
H20	0.2629	0.2761	0.2085	0.023*	
C21	0.27589 (11)	0.42735 (12)	0.26475 (7)	0.0185 (3)	
H21	0.2696	0.4791	0.2269	0.022*	
C22	0.32280 (10)	0.43990 (11)	0.45765 (6)	0.0160 (2)	
C23	0.00444 (18)	1.02239 (16)	0.41719 (9)	0.0399 (4)	
H23A	0.0692	1.0778	0.4342	0.060*	
H23B	0.0330	0.9416	0.4271	0.060*	
H23C	−0.0610	1.0378	0.4398	0.060*	

### Atomic displacement parameters (Å^2^)

**Table:**

	*U*^11^	*U*^22^	*U*^33^	*U*^12^	*U*^13^	*U*^23^
Cl1	0.03162 (17)	0.01666 (15)	0.01446 (16)	−0.00054 (11)	0.00213 (12)	0.00079 (11)
Cl2	0.02616 (16)	0.01604 (15)	0.01504 (16)	0.00147 (11)	−0.00022 (11)	0.00010 (10)
O1	0.0312 (5)	0.0167 (4)	0.0155 (5)	−0.0051 (4)	0.0004 (4)	−0.0002 (3)
O2	0.0343 (5)	0.0167 (4)	0.0139 (5)	−0.0046 (4)	−0.0007 (4)	0.0016 (3)
O3	0.0168 (4)	0.0256 (5)	0.0181 (5)	−0.0050 (4)	0.0034 (3)	−0.0004 (4)
O4	0.0181 (4)	0.0231 (5)	0.0173 (5)	0.0043 (4)	0.0031 (3)	0.0009 (4)
O5	0.0274 (5)	0.0173 (4)	0.0162 (5)	0.0042 (4)	0.0025 (4)	−0.0016 (3)
O6	0.0302 (5)	0.0199 (5)	0.0140 (5)	0.0071 (4)	0.0017 (4)	0.0008 (4)
O7	0.0603 (9)	0.0978 (12)	0.0255 (7)	0.0453 (9)	0.0063 (6)	0.0057 (7)
N1	0.0172 (5)	0.0135 (5)	0.0141 (5)	−0.0007 (4)	0.0032 (4)	−0.0008 (4)
N2	0.0188 (5)	0.0144 (5)	0.0143 (5)	0.0003 (4)	0.0020 (4)	−0.0008 (4)
C1	0.0175 (6)	0.0169 (6)	0.0158 (6)	−0.0002 (4)	0.0031 (5)	0.0012 (5)
C2	0.0162 (5)	0.0164 (6)	0.0148 (6)	−0.0006 (4)	0.0024 (4)	−0.0007 (4)
C3	0.0234 (6)	0.0162 (6)	0.0186 (7)	−0.0003 (5)	0.0024 (5)	0.0015 (5)
C4	0.0252 (6)	0.0156 (6)	0.0227 (7)	−0.0014 (5)	0.0048 (5)	−0.0034 (5)
C5	0.0188 (6)	0.0210 (6)	0.0162 (6)	−0.0015 (5)	0.0030 (5)	−0.0047 (5)
C6	0.0152 (5)	0.0206 (6)	0.0146 (6)	0.0010 (4)	0.0025 (4)	0.0001 (5)
C7	0.0138 (5)	0.0151 (5)	0.0161 (6)	0.0001 (4)	0.0038 (4)	−0.0009 (4)
C8	0.0149 (5)	0.0144 (5)	0.0183 (6)	0.0006 (4)	0.0026 (4)	0.0008 (4)
C9	0.0208 (6)	0.0199 (6)	0.0142 (6)	−0.0021 (5)	0.0057 (5)	−0.0023 (5)
C10	0.0224 (6)	0.0230 (6)	0.0169 (7)	−0.0047 (5)	0.0056 (5)	−0.0007 (5)
C11	0.0159 (6)	0.0287 (7)	0.0233 (7)	−0.0039 (5)	0.0066 (5)	−0.0048 (5)
C12	0.0155 (6)	0.0271 (7)	0.0237 (7)	0.0014 (5)	0.0041 (5)	−0.0045 (5)
C13	0.0195 (6)	0.0198 (6)	0.0173 (7)	0.0018 (5)	−0.0004 (5)	0.0009 (5)
C14	0.0200 (6)	0.0203 (6)	0.0153 (6)	−0.0011 (5)	0.0002 (5)	−0.0007 (5)
C15	0.0190 (6)	0.0160 (6)	0.0200 (7)	−0.0010 (4)	0.0068 (5)	0.0008 (5)
C16	0.0143 (5)	0.0160 (6)	0.0170 (6)	−0.0004 (4)	0.0051 (4)	−0.0004 (4)
C17	0.0131 (5)	0.0165 (6)	0.0165 (6)	−0.0002 (4)	0.0032 (4)	−0.0011 (5)
C18	0.0160 (5)	0.0158 (6)	0.0206 (7)	0.0005 (4)	0.0039 (5)	0.0006 (5)
C19	0.0156 (5)	0.0164 (6)	0.0245 (7)	−0.0010 (4)	0.0036 (5)	−0.0040 (5)
C20	0.0180 (6)	0.0236 (6)	0.0173 (6)	−0.0027 (5)	0.0042 (5)	−0.0059 (5)
C21	0.0188 (6)	0.0208 (6)	0.0166 (6)	−0.0017 (5)	0.0054 (5)	0.0007 (5)
C22	0.0144 (5)	0.0165 (6)	0.0169 (6)	0.0001 (4)	0.0030 (4)	0.0001 (4)
C23	0.0546 (11)	0.0366 (9)	0.0302 (9)	0.0102 (8)	0.0130 (8)	0.0026 (7)

### Geometric parameters (Å, °)

**Table:**

O1—C1	1.2203 (16)	C8—H8B	0.9900
O2—C1	1.3254 (15)	C9—C10	1.5108 (18)
O2—H2O	0.8401	C9—H9A	0.9900
O3—C11	1.4212 (16)	C9—H9B	0.9900
O3—C10	1.4227 (17)	C10—H10A	0.9900
O4—C13	1.4203 (16)	C10—H10B	0.9900
O4—C12	1.4275 (16)	C11—C12	1.502 (2)
O5—C22	1.2200 (16)	C11—H11A	0.9900
O6—C22	1.3228 (15)	C11—H11B	0.9900
O6—H6O	0.8402	C12—H12A	0.9900
O7—C23	1.384 (2)	C12—H12B	0.9900
O7—H7O	0.8401	C13—C14	1.5068 (18)
N1—C9	1.4869 (16)	C13—H13A	0.9900
N1—C8	1.4965 (15)	C13—H13B	0.9900
N1—H1A	0.9200	C14—H14A	0.9900
N1—H1B	0.9200	C14—H14B	0.9900
N2—C14	1.4901 (16)	C15—C16	1.5100 (17)
N2—C15	1.4987 (16)	C15—H15A	0.9900
N2—H2A	0.9200	C15—H15B	0.9900
N2—H2B	0.9200	C16—C21	1.3924 (18)
C1—C2	1.4918 (17)	C16—C17	1.4090 (17)
C2—C3	1.4021 (17)	C17—C18	1.3996 (17)
C2—C7	1.4122 (17)	C17—C22	1.4941 (18)
C3—C4	1.3872 (19)	C18—C19	1.3880 (19)
C3—H3	0.9500	C18—H18	0.9500
C4—C5	1.3864 (19)	C19—C20	1.3880 (19)
C4—H4	0.9500	C19—H19	0.9500
C5—C6	1.3889 (18)	C20—C21	1.3931 (18)
C5—H5	0.9500	C20—H20	0.9500
C6—C7	1.3975 (18)	C21—H21	0.9500
C6—H6	0.9500	C23—H23A	0.9800
C7—C8	1.5136 (17)	C23—H23B	0.9800
C8—H8A	0.9900	C23—H23C	0.9800
			
C1—O2—H2O	109.5	O3—C11—H11A	110.0
C11—O3—C10	112.56 (10)	C12—C11—H11A	110.0
C13—O4—C12	112.29 (10)	O3—C11—H11B	110.0
C22—O6—H6O	109.7	C12—C11—H11B	110.0
C23—O7—H7O	104.8	H11A—C11—H11B	108.3
C9—N1—C8	116.24 (10)	O4—C12—C11	108.49 (11)
C9—N1—H1A	108.2	O4—C12—H12A	110.0
C8—N1—H1A	108.2	C11—C12—H12A	110.0
C9—N1—H1B	108.2	O4—C12—H12B	110.0
C8—N1—H1B	108.2	C11—C12—H12B	110.0
H1A—N1—H1B	107.4	H12A—C12—H12B	108.4
C14—N2—C15	115.62 (10)	O4—C13—C14	106.89 (10)
C14—N2—H2A	108.4	O4—C13—H13A	110.3
C15—N2—H2A	108.4	C14—C13—H13A	110.3
C14—N2—H2B	108.4	O4—C13—H13B	110.3
C15—N2—H2B	108.4	C14—C13—H13B	110.3
H2A—N2—H2B	107.4	H13A—C13—H13B	108.6
O1—C1—O2	122.54 (12)	N2—C14—C13	108.82 (10)
O1—C1—C2	124.02 (11)	N2—C14—H14A	109.9
O2—C1—C2	113.44 (11)	C13—C14—H14A	109.9
C3—C2—C7	119.37 (12)	N2—C14—H14B	109.9
C3—C2—C1	118.83 (11)	C13—C14—H14B	109.9
C7—C2—C1	121.80 (11)	H14A—C14—H14B	108.3
C4—C3—C2	121.36 (12)	N2—C15—C16	112.35 (10)
C4—C3—H3	119.3	N2—C15—H15A	109.1
C2—C3—H3	119.3	C16—C15—H15A	109.1
C5—C4—C3	119.35 (12)	N2—C15—H15B	109.1
C5—C4—H4	120.3	C16—C15—H15B	109.1
C3—C4—H4	120.3	H15A—C15—H15B	107.9
C4—C5—C6	119.97 (12)	C21—C16—C17	118.28 (11)
C4—C5—H5	120.0	C21—C16—C15	117.67 (11)
C6—C5—H5	120.0	C17—C16—C15	124.05 (11)
C5—C6—C7	121.75 (12)	C18—C17—C16	119.79 (12)
C5—C6—H6	119.1	C18—C17—C22	118.99 (11)
C7—C6—H6	119.1	C16—C17—C22	121.22 (11)
C6—C7—C2	118.20 (11)	C19—C18—C17	120.98 (12)
C6—C7—C8	117.04 (11)	C19—C18—H18	119.5
C2—C7—C8	124.64 (11)	C17—C18—H18	119.5
N1—C8—C7	114.63 (10)	C20—C19—C18	119.37 (12)
N1—C8—H8A	108.6	C20—C19—H19	120.3
C7—C8—H8A	108.6	C18—C19—H19	120.3
N1—C8—H8B	108.6	C19—C20—C21	119.93 (12)
C7—C8—H8B	108.6	C19—C20—H20	120.0
H8A—C8—H8B	107.6	C21—C20—H20	120.0
N1—C9—C10	108.30 (10)	C16—C21—C20	121.54 (12)
N1—C9—H9A	110.0	C16—C21—H21	119.2
C10—C9—H9A	110.0	C20—C21—H21	119.2
N1—C9—H9B	110.0	O5—C22—O6	122.85 (12)
C10—C9—H9B	110.0	O5—C22—C17	123.80 (11)
H9A—C9—H9B	108.4	O6—C22—C17	113.35 (11)
O3—C10—C9	106.25 (10)	O7—C23—H23A	109.5
O3—C10—H10A	110.5	O7—C23—H23B	109.5
C9—C10—H10A	110.5	H23A—C23—H23B	109.5
O3—C10—H10B	110.5	O7—C23—H23C	109.5
C9—C10—H10B	110.5	H23A—C23—H23C	109.5
H10A—C10—H10B	108.7	H23B—C23—H23C	109.5
O3—C11—C12	108.67 (11)		
			
O1—C1—C2—C3	−166.11 (13)	O3—C11—C12—O4	63.74 (14)
O2—C1—C2—C3	13.74 (17)	C12—O4—C13—C14	171.72 (10)
O1—C1—C2—C7	13.7 (2)	C15—N2—C14—C13	176.57 (10)
O2—C1—C2—C7	−166.45 (11)	O4—C13—C14—N2	−55.46 (13)
C7—C2—C3—C4	0.7 (2)	C14—N2—C15—C16	−47.35 (15)
C1—C2—C3—C4	−179.44 (12)	N2—C15—C16—C21	108.39 (13)
C2—C3—C4—C5	−0.1 (2)	N2—C15—C16—C17	−71.88 (15)
C3—C4—C5—C6	−0.8 (2)	C21—C16—C17—C18	−2.78 (17)
C4—C5—C6—C7	0.94 (19)	C15—C16—C17—C18	177.49 (11)
C5—C6—C7—C2	−0.25 (18)	C21—C16—C17—C22	177.33 (11)
C5—C6—C7—C8	175.76 (11)	C15—C16—C17—C22	−2.41 (18)
C3—C2—C7—C6	−0.58 (18)	C16—C17—C18—C19	1.33 (18)
C1—C2—C7—C6	179.61 (11)	C22—C17—C18—C19	−178.77 (11)
C3—C2—C7—C8	−176.27 (12)	C17—C18—C19—C20	1.87 (18)
C1—C2—C7—C8	3.93 (19)	C18—C19—C20—C21	−3.56 (18)
C9—N1—C8—C7	−51.76 (14)	C17—C16—C21—C20	1.10 (18)
C6—C7—C8—N1	111.81 (12)	C15—C16—C21—C20	−179.15 (11)
C2—C7—C8—N1	−72.46 (15)	C19—C20—C21—C16	2.09 (19)
C8—N1—C9—C10	174.80 (10)	C18—C17—C22—O5	−154.04 (12)
C11—O3—C10—C9	179.84 (10)	C16—C17—C22—O5	25.86 (18)
N1—C9—C10—O3	−53.07 (13)	C18—C17—C22—O6	25.28 (16)
C10—O3—C11—C12	−175.41 (11)	C16—C17—C22—O6	−154.82 (11)
C13—O4—C12—C11	−177.50 (10)		

### Hydrogen-bond geometry (Å, °)

**Table:**

*D*—H···*A*	*D*—H	H···*A*	*D*···*A*	*D*—H···*A*
O2—H2O···Cl1	0.84	2.20	3.0316 (13)	170
O6—H6O···Cl2	0.84	2.12	2.9602 (13)	177
O7—H7O···Cl1	0.84	2.32	3.1586 (19)	174
N1—H1A···O1	0.92	2.05	2.7729 (16)	135
N1—H1A···O3	0.92	2.25	2.6809 (16)	108
N1—H1B···Cl2	0.92	2.27	3.1110 (13)	151
N2—H2A···O4	0.92	2.32	2.7245 (16)	106
N2—H2A···O5	0.92	2.04	2.7678 (16)	135
N2—H2B···Cl1	0.92	2.25	3.1157 (13)	156
C14—H14B···O7^i^	0.99	2.46	3.273 (2)	139
C8—H8B···Cg^ii^	0.99	2.88	3.6364 (16)	134

Symmetry codes:  (i) −*x*, *y*−1/2, −*z*+1/2; (ii) −*x*+1, −*y*+1, −*z*+1.
